# Development of an Assessment Tool for Vertical Accessibility in Spanish Homes

**DOI:** 10.1002/nop2.70243

**Published:** 2025-06-17

**Authors:** Estíbaliz Jiménez–Arberas, Isabel Fernández Méndez, Gemma Ruíz Varela, Feliciano Francisco Ordóñez Fernández

**Affiliations:** ^1^ Faculty Padre Ossó (Associated Center of the University of Oviedo) University of Oviedo Oviedo Spain; ^2^ Universidad Alfonso X el Sabio (UAX) Madrid Spain

**Keywords:** accessibility, adaptation, built environment, home modifications, occupational therapy and nursing

## Abstract

Universal accessibility is one of the most active lines of action among the steps to mitigate and compensate the restriction in participation and the limitation of activity in people with disabilities. Occupational therapists often work in a person's natural environment to assess and intervene in occupational performance. In addition to being a tool for evaluating the accessibility of individuals who visit community health professionals, especially nursing staff, this resource can significantly improve patient care and outreach efforts.

**Objective:**

The main aim of this study was to create and validate an instrument to measure the limitations in the activity of each person related to the vertical accessibility of their home.

**Design:**

The methodology of this work is a psychometric design. This is a process of construct validation of a vertical accessibility scale through confirmatory factor analysis.

**Methods:**

The scale construction is carried out following a content validation process, involving the participation of five expert occupational therapists, and the analysis of their contributions using the Attribute Agreement Analysis. In this process, Fleiss Kappa statistical analysis is used, and Kendal's W is included due to the utilisation of ordinal variables. For the construct validation of the tool, the most suitable process is followed, an Exploratory Factor Analysis, and subsequent Confirmatory Factor Analysis using Global Absolute Adjustment indices for all the subscales of the test. The analysis of the indicators was performed using the weighted least squares (DWLS) estimation method. An initial test of 63 items distributed into four subscales was configured based on the different determined accessibility spaces: (a) exterior areas and access to the building/living space; (b) horizontal mobility inside the building and common areas; (c) vertical mobility of the building/living spaces and (d) access to and the entrance door as well as the interior of the living spaces.

**Results:**

Once the analysis has been carried out with the absolute fit indices obtaining excellent values, the validation process is concluded and the final scale with 48 items is finalised. This validation process allows us to affirm that it is a useful and viable tool to evaluate accessibility. No public or patient contribution.

## 1 Introduction

Rehabilitation is a very active research area at present. In the field of occupational therapy, the environment serves as the foundation for intervention as it plays a central role in recovery and participation in activities of daily living. In the latest Occupational Therapy Practice Framework from the American Occupational Therapy Association (AOTA) (2020), the concept of ‘environment’ has been replaced by ‘context’ to reflect the terminology used in the World Health Organisation's International Classification of Functioning, Disability and Health (henceforth, ICF) (World Health Organisation 2001). In the classification, the environmental factors dimension relates to barriers to or facilitators of people's participation in activities of daily living. The ICF posits that a person's environment affects physical, social and attitudinal aspects of their activities and can improve or limit their level of functioning or participation in activities of daily living.

The home is the primary setting for occupations such as basic and instrumental activities of daily living, and appropriately designed housing promotes independence and occupational performance (Wahl et al. 2009). Ecological practice models have emphasised the influence of environmental and personal factors on the performance of daily occupations. The approach taken in the Person‐Environment‐Occupation‐Performance model, which is an example of an ecological model applied to occupational therapy, enables a more in‐depth analysis of people's capabilities, the factors facilitating occupation and the barriers to participation and wellbeing (Duncan 2011). The model acknowledges that the interaction between person, environment and occupation is dynamic and reciprocal. The environmental factors included in the model focus on occupational performance and range from social determinants, assistive technology and physical and natural environments to determinants of health, health education policies and social support.

In the White Book of Accessibility (Cuyás 2003) [White Paper on Accessibility], one of the four categories chosen to analyse problems and solutions relating to accessibility is the home, which is listed in the residential buildings subsection in the building category. According to the White Book, regional governments in Spain are responsible for supervising accessibility in the areas under their jurisdiction, e.g., urban planning. This includes financial support for functional adaptations to people's homes, which is used to fund assistive technology or housing adaptations, although these grants are aimed more at small‐scale construction works, renovations and adaptations as the amounts available range from €3000 to €7000. Autonomous communities are able to establish their own financial support programmes, including regional housing plans, additional grants and the National Housing Plan (Cuyás 2003).

The following instruments are used in both the scientific literature and in occupational therapy: (a) Housing Enabler Instrument (Iwarsson 1999); (b) Home and Community Environment instrument (HACE) (Keysor et al. 2005); (c) Home Assessment of Person‐Environment Interaction (HoPE) (Rousseau et al. 2013); (d) Comprehensive Assessment and Solution Process for Ageing Residents (CASPAR) (Sanford et al. 2002); (e) Home Safety Self‐Assessment Tool (HSSAT) (Tomita, Schweitzer, et al. 2014; Tomita, Saharan, et al. 2014); (f) Westmead Home Safety Assessment (Clemson et al. 1999); (g) Home Environmental Assessment Protocol (HEAP) (Gitlin et al. 2002). A number of instruments for assessing home accessibility can be found in the scientific literature and in occupational therapists' clinical practice, although these tools can be used by other professionals in the social and health care field, for example: nurses, psychologists, social workers… there are many professionals who include accessibility evaluation among their functions. According to UNE‐EN 17210:2021 ‘In order to define design parameters for an accessible and usable built environment it is necessary to consider the diversity of human abilities and characteristics, and consequent accessibility requirements of the intended users of the built environment’ (p. 22). Therefore, the target population of this manuscript is people with disabilities and older people regardless of diagnosis. The aforementioned ISO standard specifies ‘An accessible routes in the outdoor environment include footways, footpaths and other rights of way, such as pedestrian routes through a public space’ (p.54). (EU Standardisation 2021). From all of this arises the need to have tools that evaluate not only horizontal accessibility in the home itself but also vertical accessibility, and that are adapted to the context.

However, none of these instruments described have been translated into Spanish or adapted to the Spanish population. With the exception of the Housing Enabler Instrument, they do not analyse accessibility issues in isolation from the limitations and capabilities of specific individuals and their diagnoses. Therefore, there is a need for a tool to assess the accessibility of Spanish households that is sensitive to the Spanish context, which will also enable improvement actions to be taken in the strategic plans and incorporate the instrument as an outcome measure in the plans. In Spain, December 4, 2017, ended the deadline established by Royal Legislative Decree 1/2013, which approved the Revised Text of the General Law on Persons with Disabilities and their Social Inclusion (BOE 2024) to make structural modifications and thus make buildings under horizontal property regime accessible to all neighbours. However, there are many homes that do not comply with these regulations, so having this tool available can improve the indicators and provide current accessibility data together with the Home Environmental Scale of Accessibility II (Jiménez‐Arberas et al. 2024).

Accordingly, the main aim of this study was to create and validate an instrument to measure the limitations in the activity of each person related to the vertical accessibility of their home.

## 2 Methodology

### 2.1 Design

This paper describes a psychometric study design. This study presents the process of constructing a scale to measure accessibility using the Attribute Agreement Analysis and the construct validation process through Confirmatory Factor Analysis (CFA) of the created scale. For construct validation, absolute fit indices have been used, and incremental fit indices are added to improve the proposed model in relation to a possible base model; these are the CFI and the GFI (McNeish et al. 2018).

### 2.2 Sample

An ex post facto, non‐probability sample was used, with the final sample for analysis comprising 156 people from the Principality of Asturias. Of the total sample, 67.3% are women, concentrating by age those over 65 years old 46.79%; the rest are distributed as 23.06% under 35 years old and the range from 35 to 64 years old 30.15%. The majority are married (41.29%), single and separated 29.67%, and widowed 27.9%. The economic level is mostly between 1000 and 2000€ (32.69%), a cumulative percentage of 59.34% up to 2000€ of income. 70.32% live in apartments and 4% in single‐family houses. Regarding the location of the home, they are distributed almost equally: 57.41% are in rural areas and 42.59% are in urban areas. However, the characteristics of the homes are mostly apartment buildings (70.32%); the rest are single‐family homes.

Recruitment was made through in‐person and/or virtual interviews at different NGOs, social centres and municipalities, and once the contact was made, the assessment was carried out. For this purpose, a work team was previously trained in the use of the tool. For the definitive structure of the scale, content validation is carried out prior to construct validation and is complemented with a pilot test. In the pilot phase, the sample was composed of 20 subjects, randomly selected from the total sample.

### 2.3 Procedure

This instrument is part of an accessibility assessment model divided into two independent areas. The aim was to ensure that the scale includes the necessary characteristics to allow the different subscales in the instrument to be measured using quality standards. This validation opens up various possibilities by creating an item‐based instrument that identifies the potential obstacles affecting people's daily lives.

To develop the HESA scale, it was carried out in several phases. The first phase of these was the construction of the preliminary items for which 8 meetings were held with the team of experts (*n* = 8) where it was agreed to determine the variables that they wanted to study in relation to the vertical accessibility of the home (*n* = 5 occupational therapists; *n* = 1 psychologist, *n* = 1 quantity surveyor and *n* = 1 social worker). These four spaces were decided based on the elements commonly used in the architecture of homes in Spain and taking Decree 37/2003, of May 22, as a reference (EU Standardisation 2021). Following these meetings, the first version was achieved, comprising a total of 63 items.

The second phase was a validity analysis among judges that was conducted, and through Attribute Concordance Analysis, the framing of the items in each specific space was confirmed. This process, understood as a content validity test, allowed for the elimination of items that did not reach a concordance value. The definitive test was achieved with 63 items, which would undergo construct validity through CFA. The 5 expert (profiles different from the initial one) judges involved included researchers, educators, and clinicians in the field of disability and older adults, as well as in universal accessibility and design for all individuals.

Four specific spaces are identified in the scale: (a) exterior and building entrance; (b) horizontal mobility inside building and common areas; (c) vertical mobility inside building and (d) access to home interior. An initial questionnaire was designed using a Likert‐type scale (Likert 1932) with four response options [(1) no/never; (2) almost never; (3) often; (4) yes/always] and 63 items. This initial scale was developed with input from five occupational therapists specialising in accessibility. During this phase, the method used to develop the instrument was attribute agreement analysis (Aiken 2003), which ascertains the degree of agreement between several people making a judgement. Attribute agreement analysis allowed the uniformity of the responses given by the group of evaluators to be assessed. The statistical measure used was Fleiss' Kappa, with values over 0.75 indicating good agreement and values under 0.40 removed (Picado‐Alvarado 2008). This resulted in a reduction from the initial 63 items to 48 definitive ones.

The third phase corresponds to the pilot study with the first version of the instrument. For this purpose, various entities were contacted (NGOs, social centres, students and municipalities), the project was explained, and the participants were informed about it. Once contact was made with different entities, those people who wanted to participate signed the informed consent and after this, data collection was carried out by means of individual interviews, lasting between 30 and 90 min, depending on the characteristics of the participants at their home.

The study was approved by the Bioethics Committee (Redacted) of the Principality of Asturias (number 2020.091). In the questionnaire provided to the participants, the first question was in relation to consenting to the publication of the data anonymously, following the current Organic Law 3/2018, of December 5, on the Protection of Personal Data and Guarantee of Digital Rights.

### 2.4 Instrument

Once the initial scale had been drawn up, a pilot study was carried out with 20 randomly selected participants. The difficulties, limitations and suggestions made by the experts and the pilot group were taken into consideration when drafting the items for the final questionnaire, which was then administered to the study sample. In this way, the items were all selected in the same manner and it was possible to obtain a more accurate interpretation of the measures in the final questionnaire (Livacic‐Rojas et al. 2010; Muñiz and Fonseca‐Pedrero 2019).

### 2.5 Data Analysis

The consistency of the final questionnaire was analysed using Cronbach's α and McDonald's ω (ordinal reliability) for each of the subscales in the instrument. It has been chosen to include Cronbach's reliability due to its widespread use, although in ordinal variables the use of McDonald's omega is more appropriate, as it overcomes the limitations of alpha that require tau‐equivalent items (Cho and Kim 2015; Green and Yang 2024).

The validation process was divided into two phases. All items were analysed using exploratory factor analysis (EFA) and checked via CFA using the JASP data analysis program (JASP Team 2023). This software provides the proportion of variance for each of the factors, as well as using polychoric correlations, which are the most suitable for Likert‐type questionnaires (Ferrando and Lorenzo‐Seva 2016; Lorenzo‐Seva and Ferrando 2013).

Diagonally weighted least squares (DWLS) estimation was the method used for adjustment. This method is recommended when the assumption of normality does not hold and the sample size is not large (Freiberg Hoffmann et al. 2013). For these models, DWLS proved more robust than maximum likelihood estimation (MLE) or unweighted least squares (ULS) (Li 2016; Lloret et al. 2017). The CFA was adjusted using global or absolute fit indices for all scales of the test, which are considered the strongest (Montaño Armendariz 2022; Rojas‐Torres 2020).

- The chi‐square test, where it was estimated that values should exceed 0.05. This indicator is very sensitive as it follows a normal distribution χ^2^ (Byrne 1998; García et al. 2011), so it is advisable to supplement the results with other goodness of fit indices, such as the RMESA index, which is one of the most widely recognised (Byrne 1998; García et al. 2011; Cea 2004).
- The root mean square error of approximation (RMESA) index, in which scales with values below 0.05 are deemed valid (Browne 2022).
- The goodness of fit index (GFI), which highlights the variability explained by the model, values exceeding 0.90 are considered a good fit (Jöreskog 2022).
- The normed fit index (NFI), where values close to 1 are recommended (Bentler and Bonett 1980).
- The comparative incremental fit index (CFI), which indicates a good fit for values close to 1 and exceeding 0.95 (Bentler and Bonett 1980).

## 3 Results

### 3.1 Home Environmental Scale of Accessibility (HESA)—Exterior and Building Entrance (EBE)

The results of the exploratory factor analysis (EFA) for the Home Environmental Scale of Accessibility in Subscale 1: Exterior and building entrance produced a total of three factors.

**FIGURE 1:**
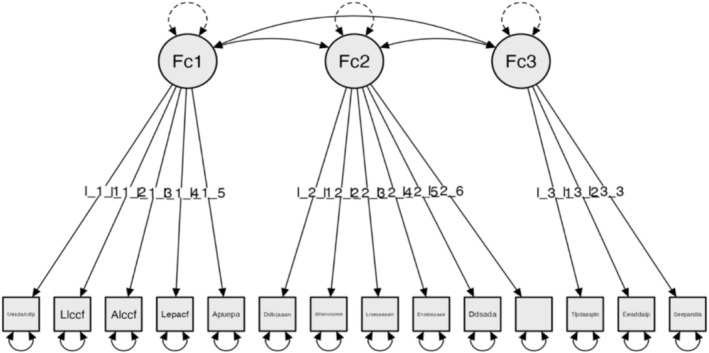
Model for Subscale 1: Exterior and building entrance (EBE).

The initial configuration comprised 16 items. Following the initial exploratory factor analysis (EFA) using the JASP program (2023) (JASP Team 2023) the result was three components with 14 items exceeding 0.4 variance. These results were used to perform a CFA, which produced the following results for the indicators:

CFA using JASP v. 0.11.1, 2019. Result of global or absolute fit indices: Chi‐square χ^2^ (74) = 48.531, *p* = 0.990; RMSEA ≤ 0.001; GFI = 0.976; NFI = 0.905 and CFI = 0.999 (Figure 1).

### 3.2 Model for Subscale 1: Exterior and Building Entrance (EBE)

The factors are configured as follows:

#### 3.2.1 Factor 1: Building Entrance Door

- The user uses the door opening/closing system.
- The user can find the lock easily.
- The user can reach the lock easily.
- The user can find the intercom easily.
- The user can reach and use the intercom.

#### 3.2.2 Factor 2: Route From Vehicle to Building

- There is an accessible route for the user from the nearest public transport stop to the building.
- Steps must be climbed to access the building.
- There is a handrail suited to the user's needs.
- A ramp or other alternative to steps is available.
- The ramp or alternative system meets the user's needs.
- There is an alternative opening system.

#### 3.2.3 Factor 3: Size of Building Entrance

- The entrance door is wide enough for the user's needs.
- There is sufficient space after opening or closing the door.
- The entrance is level with the pavement.
- Reliability analyses were performed for Subscale 1: Exterior and building entrance (EBE) and the Cronbach's α and McDonald's ω values were α = 0.780 and ω = 0.785 [0.710–0.835]. The McDonald interval was incorporated as the items were measured on an ordinal scale.

### 3.3 HESA (Horizontal Mobility Inside Building and Common Areas)

The exploratory factor analysis for Subscale 2: Horizontal mobility inside building and common areas (HMIC) obtained a total of three factors.

**FIGURE 2:**
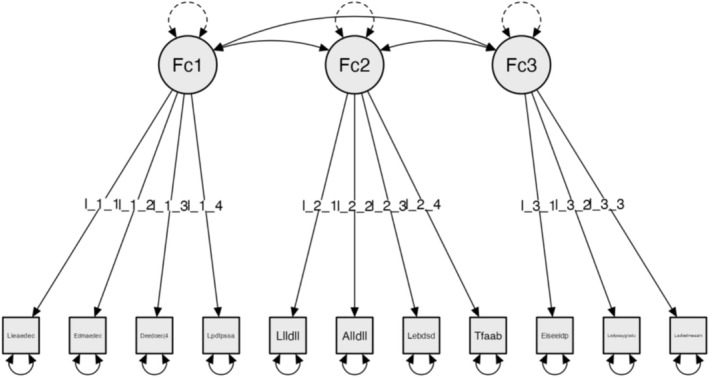
Model for Subscale 2: Horizontal mobility inside building and common areas (HMIC).

The initial configuration comprised 14 items. Following the initial exploratory factor analysis (EFA), the result was three components with 11 items exceeding 0.4 variance. These results were used to perform a CFA, which produced the following results for the indicators:

CFA, adjusting the model using the global or absolute fit indices: Chi‐square χ^2^ (41) = 46.023, *p* = 0.272; RMSEA = 0.042; GFI = 0.998; NFI = 0.800 and CFI = 0.965 (Figure 2).

### 3.4 Model for Subscale 2: Horizontal Mobility Inside Building and Common Areas (HMIC)

The factors are configured as follows:

#### 3.4.1 Factor 1: Common Areas

- The corridors on the floors are wide enough.
- The building has additional common areas.
- There is adequate lighting in the common areas.
- There are adequate mobility spaces in the common areas.

#### 3.4.2 Factor 2: Vertical Elements

- The user can find the light switches.
- The user can reach the light switches.
- The user can find the mailbox for their residence.
- The user can easily access the mailbox.

#### 3.4.3 Factor 3: Horizontal Elements

- The size of the mobility spaces meets the user's needs.
- The flooring in the entrance is adequate and is safe for the user.
- There is adequate lighting inside the entrance.
- The reliability analyses for the final version of Subscale 2: Horizontal mobility inside building and common areas (HMIC) showed Cronbach's α = 0.762 and McDonald's ω = 0.767 [0.704–0.832].

### 3.5 HESA (Vertical Mobility Inside Building)

**FIGURE 3:**
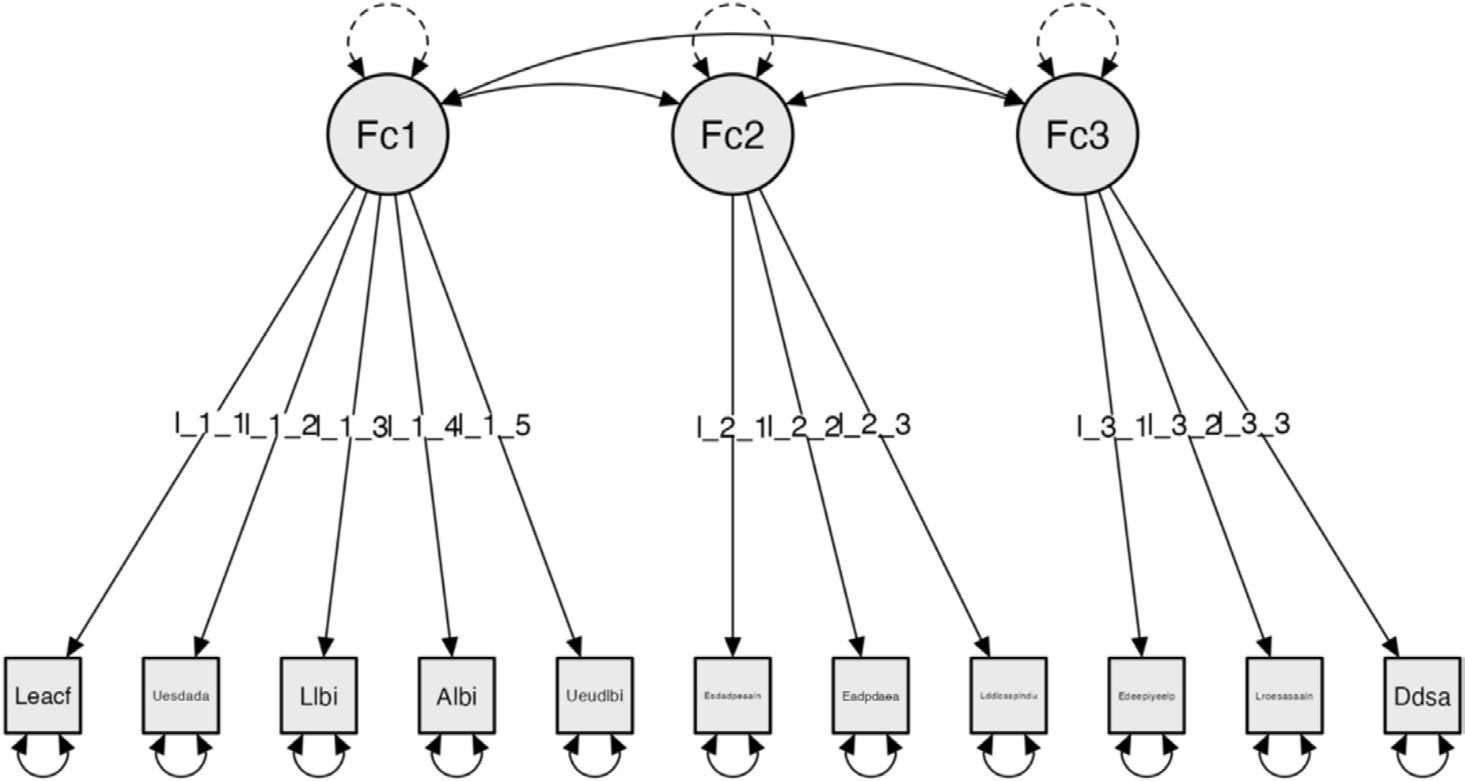
Model for Subscale 3: Vertical mobility inside building (VMIB).

The exploratory factor analysis (EFA) for Subscale 3: Vertical mobility inside building (VMIB) resulted in a total of three factors. The initial configuration comprised 15 items and the result was three components with 11 items exceeding 0.4 variance. These results were used to perform a CFA, which produced the following results for the indicators:

CFA, adjusting the model using the global or absolute fit indices: Chi‐square χ^2^ (41) = 14.452, *p* = 0.999; RMSEA ≤ 0.001; GFI = 0.999; NFI = 0.934 and CFI = 0.999 (Figure 3).

### 3.6 Model for Subscale 3: Vertical Mobility Inside Building (VMIB)

The factors are configured as follows:

#### 3.6.1 Factor 1: Location and Use of Lift

- The user uses the lift call system.
- The user can find the interior/exterior button.
- The user can reach the interior/exterior button.
- The user makes use of the interior/exterior button.
- The user can find the lift easily.

#### 3.6.2 Factor 2: Entrance and Space in Lift

- The door opening system meets the user's needs.
- The width of the lift door is adequate.
- The size of the lift cabin meets the user's needs.

#### 3.6.3 Factor 3: Lift‐to‐Floor Transition

- The lift's interior is not at the same level as the floor when it stops.
- An alternative system is available.
- The ramp or alternative system meets the user's needs.
- The reliability analyses for the final version of Subscale 3: Vertical mobility inside building (VMIB) showed Cronbach's α = 0.700 and McDonald's ω = 0.702 [0.647–0.816].

### 3.7 HESA (Access to Home Interior)

The exploratory factor analysis (EFA) for Subscale 4: Access to home interior (AHI) also obtained three factors.

**FIGURE 4:**
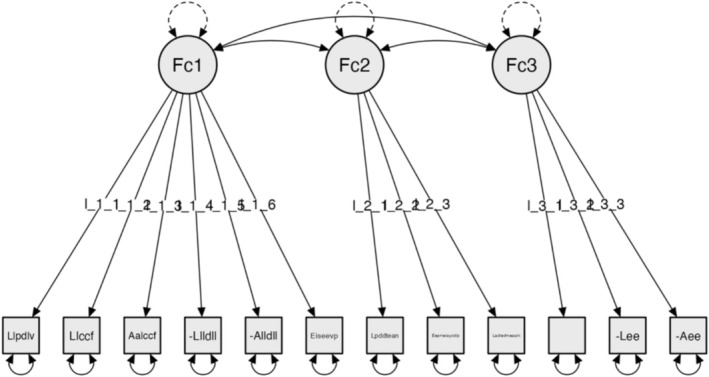
Model for Subscale 4: Access to home interior (AHI).

The initial configuration comprised 18 items. Following the initial analysis, the result was three components with 12 items exceeding 0.4 variance. These results were used to perform a CFA, which produced the following results for the indicators:

CFA, adjusting the model using the global or absolute fit indices: Chi‐square χ^2^ (51) = 17.493, *p* = 0.999; RMSEA ≤ 0.001; GFI = 0.999; NFI = 0.948 and CFI = 0.999 (Figure 4).

### 3.8 Model for Subscale 4: Access to Home Interior (AHI)

The factors are configured as follows:

#### 3.8.1 Factor 1: Access to Home Entrance

- The user can find the door to their home.
- The user can find the lock easily.
- The user can reach the lock easily.
- There is adequate lighting in the hall/corridor.
- The user can find the light switches.
- The user can reach the light switches.

#### 3.8.2 Factor 2: Manoeuvrability of Home Entrance

- The door to the home is wide enough.
- There is room to manoeuvre when opening and closing the door.
- The size of the mobility spaces meets the user's needs.

#### 3.8.3 Factor 3: Use of Furniture in Home Entrance

- There is furniture inside the home entrance to facilitate the user's needs (removing shoes, putting down objects, hanging coats, etc.).
- The user can find these elements.
- The user can reach these elements.
- The reliability analyses for the final version of Subscale 4: Access to home interior (AHI) showed Cronbach's α = 0.866 and McDonald's ω = 0.871 [0.787–0.918].
- At the end of this process, the final scale comprised four subscales with a total of 48 items. Each of the four subscales encompasses three factors.

## 4 Discussion

In this study, a scale for assessing accessibility in relation to individuals and their daily occupations, which is suitable for use outdoors and in the immediate vicinity of the home, was designed and validated. The study shows that vertical accessibility to the building, which is legislated in our Autonomous Community (LIONDAU, n.d.) accessibility comprises a large number of interrelated components, with different variables that affect people's participation in daily life.

From her perspective as an architect, Knudstrup (2012) identifies the most relevant physical factors and architectural elements affecting subjective wellbeing in older adults' homes, highlighting the following as key points: location, type of residence, common areas, accessibility, interior design and decoration, technology, colours and lighting, layout and exterior spaces. Meanwhile, Bahl (2017) explains that the environment may be built in such a way as to help compensate for the capabilities lost by an individual and improve those that they have maintained. Current strategic plans for elderly people include interventions to keep them living at home for longer (Allen et al. 2019).

Although there are various assessment instruments available in the literature, none have been validated for the Spanish population or adapted to Spanish households. Moreover, there are few instruments that are not specific to a type of disability or to elderly people. The instrument developed in this study is intended to assess accessibility regardless of individual circumstances. In the context of the ICF (World Health Organisation 2001), this falls under the environmental factors dimension. By way of example, an individual may be subjected to very different factors in different homes and view them as either barriers or facilitators.

Several systematic reviews analysed the psychometric properties of validation tools for adult life activities and activity measurement instruments and concluded that reliability is the most demonstrated propriety. They affirm that the methodological quality of the studies does not present the psychometric properties of the measurement instruments (Echeverría et al. 2021; Patry et al. 2019) For example, in the systematic review conducted by Patry et al. (2019) regarding the most used aim measures of accessibility in the domestic environment to evaluate their psychometric properties, they concluded, after comparing the analyses of 10 studies, that no measure showed solid evidence of reliability and validity. In this line, the results of the analysis conducted on some tests highlight: Housing Enabler, where three versions from 2005, 2007, and 2010 were analysed. This test only provides content validity based on Kappa analysis. The I‐HoPE scale, in the two articles analysed from 2005 and 2010, seems to provide evidence of construct validity based on the correlation performed with the Housing Enabler test, which, as indicated by the authors of their systematic review, does not show solid evidence refuting its validity. This is referred to as concurrent validity, as it bases its validity on the validation of the test being compared. Finally, in the analysis of the HACE scale, content validity is evidenced through Kappa analysis, and a construct analysis is conducted using the chi‐square statistic. As it does not provide control statistics such as goodness of fit, this process is considered incomplete and inconclusive. This study allows us to affirm that our scale has better psychometric properties than those developed and used up to this point. The HESA instrument has included the entire construct validation process, in addition to reliability with its psychometric properties, thus surpassing the criteria of rigour of studies on validity and systematic reviews of different measurement scales related to daily activity and accessibility.

The HESA serves to assess home accessibility based on the person assisted and not based on a diagnosis. Based on this assessment, health professionals will be able to design intervention strategies at the user's own environment, adapted to their health conditions or participation in their occupations. Likewise, specific intervention strategies can be designed for the user himself that will be directly related to his own environment, with which his level of participation will benefit either through restorative strategies towards the client's own factors or towards the demands of his environment.

This is largely what differentiates it from other instruments described above, such as HoPE, which is aimed at people with physical disabilities, I‐HOPE Assist, which is aimed at carers, and HACE, which is aimed at elderly people. Although the Housing Enabler Instrument has the same objective as our instrument, it has not been adapted to the Spanish context, lacks the excellent psychometric properties of other instruments, and requires the use of a specific program that limits its application in clinics and public institutions. Both I‐HOPE and HACE have reliability values within the acceptable range for their different subscales, but Cronbach's alpha was used to confirm these values. In this study, ordinal reliability was also used as it is more appropriate when using items with interval measures (Ferrando and Lorenzo‐Seva 2016; Lorenzo‐Seva and Ferrando 2006). This aspect is not covered by other scales currently in use.

As stated above, the most widely recommended indicator, DWLS, was used as it is best suited to these types of items (Li 2016; Lloret et al. 2017). When items are measured in this way, polychoric correlation matrices must be used, as is the case in our study.

Given all these considerations, the results of the psychometric and confirmatory factor analyses performed for this study allow us to confirm that the final scale has an adequate factor structure, reliability and validity.

The reliability analyses for the subscales were acceptable in all cases. Cronbach's α was above the minimum recommended value of 0.70 (Huh et al. 2006), with the range of validity between 0.7 and 0.8. As for ω (ordinal alpha, McDonald's ω), an acceptable level of reliability is deemed to be between 0.70 and 0.90 (Campo‐Arias and Oviedo 2008). The results obtained in the CFA for the final questionnaire reduced to 48 items were excellent in all indicators, confirming that the HESA has robust construct validity.

In the CFA, the standard procedure was followed. A principal component analysis was first carried out to help us analyse a data set without any kind of prior hypothesis as to the structure and the results of this analysis provided the model (Pérez 2020) This analysis allowed us to establish an initial structural hypothesis. The Likert‐type response options used in the questionnaire allowed the software to use polychoric correlation matrices to establish the final number of factors and items.

Among the limitations of the study, there are several biases to point out in the present manuscript, the first of which is the sampling bias, since the participants are volunteers and it is a convenience sample, and all the participants belong to the same autonomous community (Principality of Asturias), so the instrument was not applied in other autonomous communities.

The demographic characteristics of the study sample—primarily individuals over the age of 65 (54%), predominantly women (63.3%), with a mid‐range socio‐economic status (EUR 1000–2000; 42.25%), residing largely in urban central areas (52.82%; 16.2%) and living in apartment blocks (67.6%)—may limit the generalisability of the findings. This demographic concentration introduces a potential sampling bias, reducing the applicability of the results to broader populations, particularly those in rural areas, younger age groups or those living in different housing environments. Consequently, while the findings may be relevant and insightful for urban, ageing populations with similar socio‐demographic profiles, caution is advised when extrapolating conclusions to more diverse or underrepresented segments of the population. However, older adults represent a particularly relevant population when exploring issues related to functioning, participation and environmental accessibility, in line with the International Classification of Functioning, Disability and Health (ICF). In this age group, limitations in activity and restrictions in participation are frequently observed due to physical, cognitive, and mental health conditions, which often coexist and intensify the complexity of their daily functioning.

From a care perspective, older individuals are frequently supported within domestic environments rather than institutional settings. This home‐based care context is of critical importance, as it underscores the need to examine and adapt living environments to support autonomy and quality of life in ageing populations.

Moreover, although a high percentage of older participants in the study reside in apartment buildings, this type of housing is among the most prevalent in Spain. Therefore, the predominance of this housing form does not reflect a selection bias but rather mirrors national demographic and urban planning trends. It is also worth noting that the functional suitability of housing for older adults is less dependent on typology per se, and more influenced by vertical and horizontal accessibility—features which directly impact mobility, independence, and participation.

Thus, including older adults as the primary sample enhances the ecological validity of the study, providing insights that are both demographically representative and highly relevant for guiding interventions in the field of ageing, rehabilitation, and assistive environments.

In future research, the sample size will be increased for some of the descriptive variables by including more age groups, different limitations in activity or restriction in participation and different types of residence, for example, to improve the robustness of the test. The instrument will also be applied to different populations and adjusted accordingly. Another line of future research is to incorporate this tool for the assessment of outcomes to improve current policies on accessibility, since despite the efforts invested, greater specificity is needed in homes. Likewise, this tool can be used by different professionals in the socio‐health field such as occupational therapists to help reduce subjectivity to contribute to decision‐making. Finally, it should be noted as a limitation that this initial study focuses on the validation of the scale, and it would be advisable that as the data is utilised, it enables a grading process to be implemented.

Finally add that the exploratory and confirmatory factor analyses carried out for this validation process were done with a single sample.

## 5 Conclusion

Occupational therapists are experts when it comes to the relationship between individuals and the environment(s) where they carry out activities of daily living. Most occupations, especially activities of daily living and instrumental activities of daily living, are performed at home. In parallel, this type of tool can benefit other health professionals working with people with disabilities and working directly on accessibility and people and increase awareness of Design for All in higher education institutions in Europe such as the European Design for All e‐Accessibility.

In conclusion, the HESA scale is useful as an initial accessibility scale and has adequate validity indices, making it appropriate for use by occupational therapists and other social‐health professionals.

### 5.1 Addendum

Due to the amount of data used to produce this article, a supplementary file has been compiled and is available at https://cutt.ly/6N0P6S9.

Supplementary file contains: Results of the reliability analyses by item for the Accessibility subscales. Descriptive statistics for the items, correlation data and reliability analyses using McDonald's ω and Cronbach's α.

## Author Contributions

Dr. Estíbaliz Jimenez Arberas and Prof. Isabel Fernández have been working and teaching in the Occupational Therapy program at Padre Ossó Faculty of the University of Oviedo for several years. During this time, they have been studying and analysing the accessibility issues faced by individuals, concluding that it is necessary to develop a scale that would allow for a free and easy assessment of the difficulties individuals may encounter in the various spaces where they carry out their daily activities. Based on this, they have applied their knowledge and experience to drafting the scale and selecting the items. On the other hand, Dr. Gemma Ruíz Varela has conducted her research activities in the field of methodology and education at Alfonso X el Sabio University. Her contribution to the research process has focused on drafting and the initial analysis of the scale. Dr. Feliciano, as a professor of statistics and methodology and Research Coordinator at the Faculty of Education Sciences at Alfonso X el Sabio University, has conducted all data analysis in this work, as well as designing the research necessary for the validation process and drafting the different stages of the creation process and the final results of the scale. Finally, all authors have reviewed the article in terms of its content and writing, resulting in the article that has been submitted.

## Conflicts of Interest

The authors declare no conflicts of interest.

## Data Availability

The data that support the findings of this study are available from the corresponding author upon reasonable request.
